# Active left atrial emptying: assessment by cine and velocity encoded magnetic resonance imaging

**DOI:** 10.1186/1532-429X-13-S1-P257

**Published:** 2011-02-02

**Authors:** Kai Muellerleile, Michael Groth, Dennis Saering, Daniel Steven, Arian Sultan, Imke Drewitz, Boris Hoffmann, Gerhard Adam, Gunnar K Lund, Stephan Willems, Thomas Rostock

**Affiliations:** 1 University Medical Center Hamburg-Eppendorf, Hamburg, Germany

## Purpose

To compare the ability to assess active left atrial (LA) emptying between cine- and velocity encoded (VENC) magnetic resonance imaging (MRI).

## Introduction

Growing attention has been recently paid to the assessment of active LA emptying in patients after ablation of atrial fibrillation. Measurements of active LA emptying are typically performed by echocardiography in clinical routine. However, little is known about the role of cardiac MRI for this purpose. The present study evaluated the relative merits of biplane long-axis cine- and VENC-MRI for the assessment of active LA emptying.

## Methods

**Figure 1:**
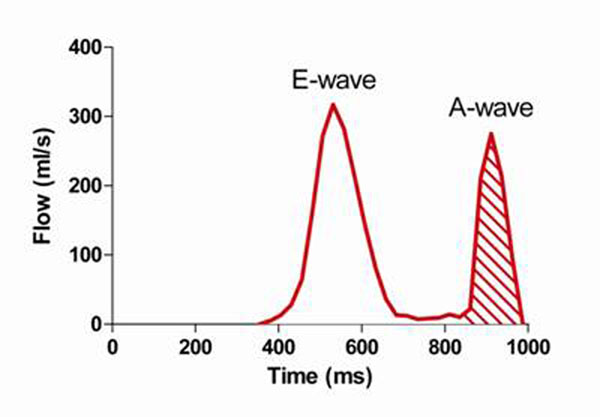
Assessment of active LA emptying volume by VENC-MRI

This study included 82 patients with sinus rhythm. Cine-MRI was performed in the four- and two chamber view. Maximal, mid-diastolic and minimal LA volumes were calculated using the biplane area-length method. Active LA emptying volume was defined by cine-MRI as the difference between mid-diastolic and minimal LA volume. Transmitral flow was assessed by VENC-MRI. Flow curves were generated using the freely available software Segment. Active LA emptying was defined as the volume of late diastolic flow (shaded area under the A-wave, Figure 1). Correlation and agreement was assessed between cine- and VENC-MRI measurements of LA emptying volumes. Reproducibility was evaluated by calculating the intra- and inter-observer variances.

## Results

A strong correlation was found between biplane long-axis cine- and VENC-MRI measurements of active LA emptying volume (r=0.72, P<0.0001). Bland-Altman analysis revealed a mean difference of 3.4±9.1 ml between both methods. The intra- and inter-observer variances were significantly larger for measurements by cine-MRI compared to measurements by VENC-MRI (22.5 ml2 vs. 1.6 ml2 and 45.5 ml2 vs. 5.8 ml2; P<0.0001).

## Conclusions

Measurements of active LA emptying agree well between biplane long-axis cine- and VENC-MRI. However, measurements by VENC-MRI provide a significantly better reproducibility and should preferably be used.

